# Neuro-psychiatric co morbidity in HIV/AIDS; and effect of alcohol in CD4 count in HIV patients

**DOI:** 10.1186/1471-2334-14-S3-E26

**Published:** 2014-05-27

**Authors:** Biswadeep Borthakur, Dhruba Jyoti Borah, Supria Kumar Mandal, Kamala Deka, Saumitra Ghosh, Dhruba jyoti Bhuyan, BB Rewari, D Singh

**Affiliations:** 1 Assam Medical College, Dibrugarh, Assam, India; 2 National AIDS Control Organisation, New Delhi, India

## Background

The exponential increment of various co-morbid disorders among HIV positive individuals includes various medical subspecialties and mental health is one of them. HIV/AIDS affects mental health by its complex interactions which may be the result of the psychological response to the diagnosis or complex neurobiological interactions. Therefore the current study aims at systematic evaluation of psychiatric morbidity in HIV/AIDS patients.

## Methods

Patients attending ART centre during the period of 1 year were selected for the study by random sampling method. After obtaining written consent MINI-PLUS intentional scale was applied to evaluate the psychiatric co-morbidity.

## Results

Of 75 patients MINI-Plus interview revealed co-morbidities of major depressive disorder (10.66%), generalized anxiety disorder (25.33%), panic disorder (1.33%), alcohol dependence (29.33%) and abuse (5.33%), nicotine dependence (32%), opioid & marijuana (1.33%) and schizophrenia (2.66%) and cognitive impairment (5.33%). Both the sex were almost equally affected by the psychiatric morbidities except substance abuse were more prevalent in male participants but patients with primary and secondary education suffered more from anxiety disorder. Interestingly, patients with co-morbid alcohol abuse and dependence had lower CD4 count than non alcoholic counterpart (*P* = 0.0426).

## Conclusion

HIV/AIDS with psychiatric morbidity, out of which generalized anxiety disorder and major- depressive- disorder are common but alcohol and nicotine dependence and abuse, is the highest of co-morbidity. Co-morbid alcohol use problem is associated with low CD4 probably mediated through immune-modulator effect of alcohol. Treatments of co morbidity will definitely ensure a better quality of life and treatment outcome.

